# Analysing culture methods of frozen human ovarian tissue to improve follicle survival

**DOI:** 10.1530/RAF-20-0058

**Published:** 2021-03-23

**Authors:** Briet D Bjarkadottir, Charlotte A Walker, Muhammad Fatum, Sheila Lane, Suzannah A Williams

**Affiliations:** 1 Nuffield Department of Women’s and Reproductive Health, University of Oxford, Oxford, UK; 2 Oxford Fertility, Institute of Reproductive Sciences, Oxford, UK; 3 Department of Paediatric Oncology and Haematology, Oxford University Hospitals NHS Foundation Trust, Oxford, UK

**Keywords:** cryopreservation, ovary, follicle, human, in vitro growth, fertility preservation

## Abstract

*In vitro* follicle growth is a potential fertility preservation method for patients for whom current methods are contraindicated. Currently, this method has only been successful using fresh ovarian tissue. Since many patients who may benefit from this treatment currently have cryopreserved ovarian tissue in storage, optimising *in vitro* follicle growth (IVG) for cryopreserved-thawed tissue is critical. This study sought to improve the first step of IVG by comparing different short-term culture systems for cryopreserved-thawed human ovarian tissue, in order to yield a higher number of healthy multilayer follicles. We compared two commonly used culture media (αMEM and McCoy’s 5A), and three plate conditions (300 µL, 1 mL on a polycarbonate membrane and 1 mL in a gas-permeable plate) on the health and development of follicles after 6 days of culture. A total of 5797 follicles from three post-pubertal patients (aged 21.3 ± 2.3 years) were analysed across six different culture conditions and non-cultured control. All culture systems supported follicle development and there was no difference in developmental progression between the different conditions tested. Differences in follicle morphology were evident with follicles cultured in low volume conditions having significantly greater odds of being graded as morphologically normal compared to other conditions. Furthermore, culture in a low volume of αMEM resulted in the highest proportion of morphologically normal primary and multilayer follicles (23.8% compared to 6.3-19.9% depending on condition). We, therefore, recommend culturing cryopreserved human ovarian tissue in a low volume of αMEM to support follicle health and development.

**Lay summary:**

Ovaries contain a large number of follicles, each containing an immature egg and other important cells. Cancer treatments can lead to long-lasting negative side effects to the ovaries including the destruction of follicles, resulting in infertility. One strategy to preserve fertility is freezing of ovaries or ovarian tissue in girls and women undergoing cancer treatment. The long-term aim is to thaw and grow their ovarian tissue in the laboratory to obtain mature eggs, which can then be fertilised. In this study, we compared six different methods of growing previously frozen human ovarian tissue in order to best support follicle growth and health. We found that using the lowest amount of αMEM medium (a specific type of nutrient-rich growth solution) resulted in the highest proportion of healthy follicles. Improving the methods used to grow ovarian tissue, particularly frozen tissue, is important for future fertility preservation.

## Introduction

Advances in cancer treatment have led to increased survival rates, particularly among young children and adolescents. The increased number of young cancer survivors highlights the need for effective fertility preservation methods for these individuals. Current methods of female fertility preservation include cryopreservation of eggs, embryos and ovarian tissue (Wallace 2011, Yasmin *et al.* 2018). However, pre-pubertal girls are unable to undergo egg or embryo cryopreservation and for some of these patients, reimplantation of ovarian tissue is contraindicated due to the risk of reintroducing malignant cells (Bastings *et al.* 2013, Rosendahl *et al.* 2013). There is therefore a need to develop alternative fertility preservation methods for these patients (Salama *et al.* 2019).

One potential method of fertility preservation is *in vitro* growth (IVG) of follicles by culturing fresh or frozen ovarian tissue followed by *in vitro* maturation (IVM) of immature oocytes resulting in mature developmentally competent eggs (Salama & Woodruff 2019, Telfer 2019). This was recently achieved using fresh ovarian tissue and a four-step culture protocol (McLaughlin *et al.* 2018). In the first step, small pieces of ovarian cortical tissue, each containing primordial follicles, are cultured for 6–8 days to allow for the development of multilayer follicles which can then be isolated for subsequent culture (Telfer *et al.* 2008, McLaughlin *et al.* 2018). This is a limiting step for IVG as it governs the number of follicles that can be carried on to the next stages. Thus, optimisation of the initial step of ovarian tissue culture to increase the yield of healthy multilayer follicles is critical for the success and subsequent clinical application of this method. Furthermore, since many patients who would benefit from IVG have already undergone ovarian tissue cryopreservation, it is important that IVG techniques are established for both fresh and cryopreserved tissue samples.

Since the first report of human ovarian cortical tissue culture in the 1990s (Hovatta *et al.* 1997), a number of different culture systems have been developed. These methods range from culturing tissue pieces in a low volume of medium (Telfer *et al.* 2008, McLaughlin *et al.* 2018) to culture on hydrophobic membranes (Walker *et al.* 2019, Lopes *et al.* 2020) and membrane inserts (Scott *et al.* 2004, Garor *et al.* 2009). The most commonly used basic media are McCoy’s 5A (McLaughlin *et al.* 2018, Walker *et al.* 2019, Lopes *et al.* 2020) and αMEM (Scott *et al* 2004, Huang *et al.* 2008, Garor *et al.* 2009, Isachenko *et al.* 2012, Lerer-Serfaty *et al.* 2013, Asadi-Azarbaijani *et al.* 2016, Ramezani *et al.* 2017), with the latter frequently being used for cryopreserved-thawed tissue. Although animal models have suggested that fresh and frozen ovarian tissue may have different metabolic requirements and require different media (Castro *et al.* 2014), there has been no reported comparison between these two commonly used culture media for the culture of cryopreserved human ovarian tissue. In addition, it has been reported that increased oxygen availability through culture of fresh human ovarian tissue in gas-permeable culture plates had a positive impact on follicle health and development (Talevi *et al.* 2018) and thus this is an important variable that needs to be further investigated.

In this study we sought to compare different methods of culturing cryopreserved-thawed human ovarian tissue in order to maximise follicle development and health during the initial step of IVG. Based on existing methods in the published literature, we selected six culture conditions, comparing the effects of two types of culture media and three plate conditions with similar oxygen availability. We report here the effect of each of these conditions on follicle progression and morphology after 6 days of culture, using statistical modelling to account for intra- and inter-patient variability.

## Materials and methods

### Ovarian tissue collection

The use of human tissue was approved by Health Research Authority South Central – Oxford B Research Ethics Committee (REC reference: 14/SC/0041). Cryopreserved ovarian tissue was obtained from the Oxford Cell and Tissue Biobank. Patient selection criteria included post-pubertal patients undergoing unilateral oophorectomy and subsequent ovarian tissue cryopreservation due to malignancy or blood disorder. Exclusion criteria included ovarian cancer and prior chemotherapy or radiation treatment. As part of the consent process, permission to use tissue in research had been obtained. Cortical strips were cryopreserved using slow freezing by the Oxford Cell and Tissue Biobank in 1.5 M ethylene glycol, 0.1 M sucrose and 10% serum substitute supplement in Leibovitz L-15 medium and stored in vapour phase liquid nitrogen.

### Chemicals and consumables

Leibovitz L-15 medium (11415049), minimum essential medium alpha (αMEM) (22561021), McCoy’s 5A (modified) HEPES buffered medium (22330021), l-glutamine (25030024) and ascorbic acid (10012011) were purchased from Thermo Fisher. Human serum albumin (AI653), ITS liquid media supplement (100×; I3145), Penicillin and streptomycin (P0781) sodium pyruvate (S8636), neutral red (N2889), sucrose (S7903), ethylene glycol (324558), Whatman Nucleopore membranes (WHA110414), Bouin’s solution (HT10132), Gill no 2 haematoxylin (GHS232), Eosin Y solution (HT110332) and DPX mountant (06522) were purchased from Sigma Aldrich. Recombinant human follicle-stimulating hormone (FSH; Gonal-F; Z1540) was purchased from Merck Serono (Feltham, UK). Corning Costart tissue culture treated 24-well plates were purchased from Scientific Laboratory Supplies (Nottingham, UK). Lumox® 24-well plates were generously provided by Sarstedt (Nümbrecht, Germany).

### Tissue thawing and cortical strip culture

Cortical strips were thawed and processed for culture as described previously (Walker *et al.* 2019). Briefly, following thawing in a water bath (30°C for 3 min), cortical strips were washed through thawing solutions for 5 min each at room temperature to remove cryoprotectants. The thawing solutions contained a reversed ethylene glycol gradient (1.0, 0.5 and 0 M), 0.1 M sucrose and 3 mg/mL human serum albumin (HSA) in L-15 medium. Thawed strips were transferred to dissection medium (3 mg/mL HSA, 100 U/mL penicillin, 100 µg/mL streptomycin, 2 mM l-glutamine and 2 mM sodium pyruvate in L-15 medium) and mechanically chopped using a McIlwain tissue chopper (Campden Instruments Ltd, UK), after which the fragments were further cut manually using scalpels and forceps into approximately 0.5 × 0.5 × 0.25 mm pieces. Tissue fragments were incubated in 25 μg/mL neutral red for 1 h to visualise fragments with viable follicles (Chambers *et al.* 2010, Walker *et al.* 2019).

**Figure 1:**
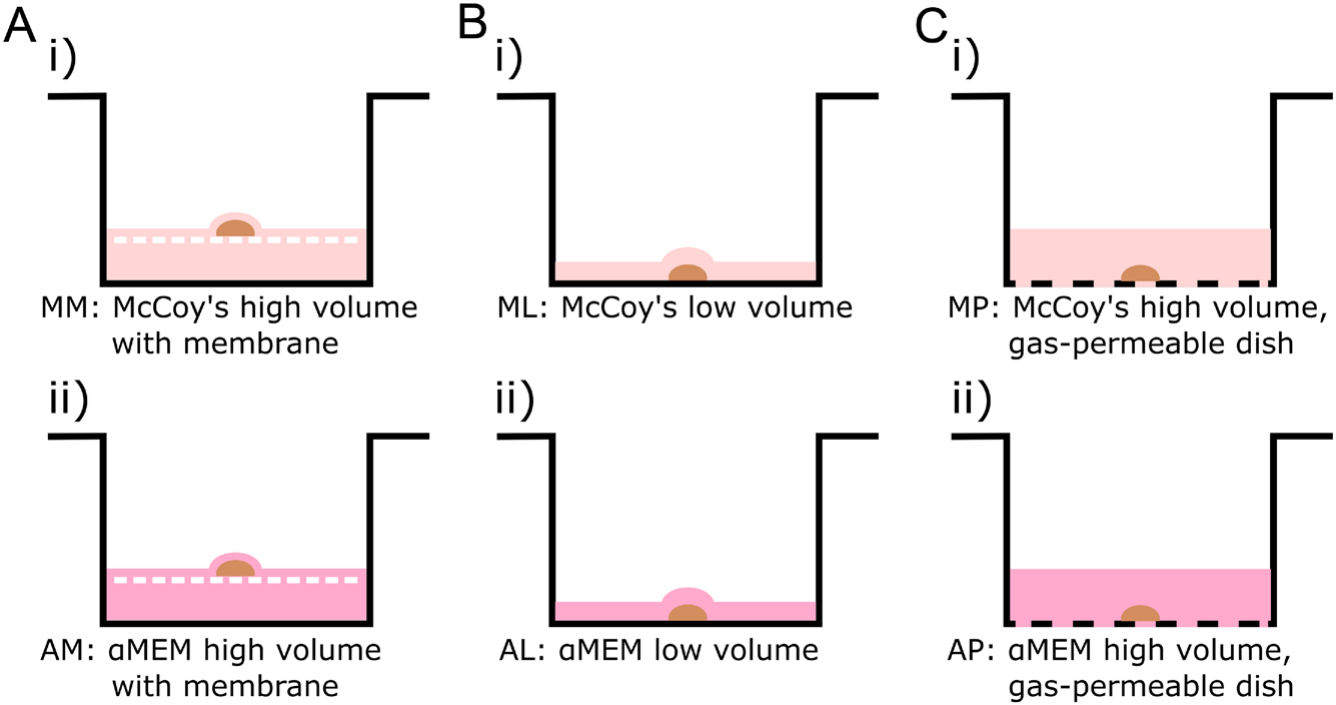
Overview of culture conditions. Cryopreserved human ovarian tissue (represented by a brown half-moon shape, not to scale) was cultured under different culture conditions. Three plate conditions were tested along with two culture media, resulting in a total of six culture conditions. (A) Polycarbonate membrane floating in a high volume (1 mL) of McCoy’s 5A (MM; Ai) or αMEM (AM; Aii) medium in a conventional 24-well plate. (B) Low volume (300 μL) of McCoy’s 5A (ML; Bi) or αMEM (AL; Bii) medium in a conventional 24-well plate; (C) High volume (1 mL) of McCoy’s 5A (MP; Ci) or αMEM (AP; Cii) medium in a gas-permeable Lumox® 24-well plate.

Tissue pieces were distributed randomly and evenly between six different culture conditions (9–12 pieces per condition per patient), fragments, where red staining was observed, were allocated before non-stained fragments. One piece of tissue was placed in each culture well. A portion of tissue was fixed overnight in Bouin’s solution as a non-cultured control. Three different plate conditions were tested: a polycarbonate membrane (13 mm diameter, 8 µm pore size) floating in 1 mL of medium in a conventional 24-well culture plate, 300 µL of medium in a conventional 24-well plate, and 1 mL of medium in a gas-permeable Lumox® 24-well plate (Fig. 1). The well size and diameter were the same for both types of culture plates and in all conditions the ovarian tissue was located at the medium-gas interface. Two different media were compared for each plate condition (i.e. six experimental conditions in total): McCoy’s 5A and αMEM, both supplemented with 1 mg/mL HSA, 100 U/mL penicillin, 100 µg/mL streptomycin, 2 mM L-glutamine, 10 µg/mL insulin, 5.5 µg/mL transferrin, 5 ng/mL selenium, 50 µg/mL ascorbic acid and 12.5 IU/L recombinant human FSH. Cortical tissue pieces were cultured for 6 days at 37°C under 5% CO_2_ in air, with medium changes every other day (half the medium removed and fresh medium added). For tissue cultured on a polycarbonate membrane, care was taken to visually confirm that the tissue pieces were covered by a thin layer of medium during the entire culture period. Following the culture period, all tissue pieces were fixed in Bouin’s solution overnight before storage in 70% ethanol at 4°C.

### Histological analysis

Fixed ovarian tissue was dehydrated in a graded series of ethanol, cleared in xylene and embedded in paraffin wax. The wax-embedded tissue was entirely serially sectioned at 5 µm, mounted on glass slides and stained with haematoxylin and eosin.

Follicles were staged as previously described (Gougeon 1996, Walker *et al.* 2019) as primordial (single layer of flattened pre-granulosa cells), transitional (single layer with at least one cuboidal granulosa cell), primary (complete layer of cuboidal granulosa cells) and multilayer (at least one complete layer of granulosa cells plus one or more partial or complete layers). Follicles were graded according to morphology based on the presence of pyknotic granulosa cells or oocyte and shrinkage of the ooplasm as previously described (Walker *et al.* 2019). Morphologically normal follicles were defined as having a non-pyknotic non-shrunken oocyte with non-pyknotic granulosa cells, degenerating follicles had one of the above factors, while follicles were classified as atretic if they had both an oocyte with a pyknotic nucleus and pyknotic granulosa cells. Every tissue section was analysed and each follicle was followed through neighbouring sections to avoid double counting. Only follicles with a visible nucleolus or a clearly defined nuclear membrane were assessed. Follicles were analysed by two independent researchers with at least 10% overlap to ensure consistency in staging and health grading.

Every 12^th^ section of each piece of tissue was imaged at a low magnification and the area measured using the freehand measuring tool in ImageJ 1.46r (National Institutes of Health, USA; Schneider *et al.* 2012, Rueden *et al.* 2017). Average area measurements were used to calculate the volume of each tissue piece. Follicle density was determined by dividing the total number of follicles counted in a tissue sample by the tissue volume. All imaging was performed using a Leica DM2500 microscope (Leica Microsystems, Germany), fitted with a QImaging Micropublisher 5.0 RTV camera running the QCapture Pro 7 software (QImaging, Canada).

### Statistical analysis and modelling

All statistical analyses were performed using R statistical software, version 3.5.0. A generalised linear mixed model following a negative binomial distribution (glmmPQL; Venables & Ripley 2002) was used to determine the effect of culture condition on follicle development, adjusted for patient and tissue volume. A proportional odds model (clmm2; Christensen 2015) was used to determine whether follicle morphology was affected by culture condition, again adjusting for tissue volume and patient. Data are presented as mean (±s.e.m.), combined proportions (%) from all patients or as odds ratios (OR) with 95% CIs, unless otherwise stated, and statistical significance was defined as *P*  < 0.05.

## Results

### Patient characteristics

Samples from three post-pubertal patients (aged 17–25 years, mean 21.3 ± 2.3 years) were used in this study. Table 1 shows patient age, diagnosis and non-cultured follicle density. The total number of follicles analysed from all three patients across all conditions was 5797. There was great variation in follicle density in both cultured and non-cultured tissue between patients, ranging from 20.4 ± 5.5 to 431.9 ± 23.8 follicles/mm^3^ for non-cultured tissue (Table 1). This, coupled with our group’s previous work (Walker *et al.* 2019), highlighted the need for statistical modelling to account for this intra-patient variability.

**Table 1:** Patient characteristics and non-cultured follicle density.

Patient	Age (years)	Diagnosis	Total follicles analysed	Non-cultured follicle density (follicles/mm^3^)
1	25	Cervical cancer	303	20.4 ± 5.5
2	17	Ewing’s sarcoma	3198	431.9 ± 23.8
3	22	Atypical teratoid rhabdoid tumour	2295	127.7 ± 77.3

### Follicle development was predominantly unaffected by culture condition

**Figure 2:**
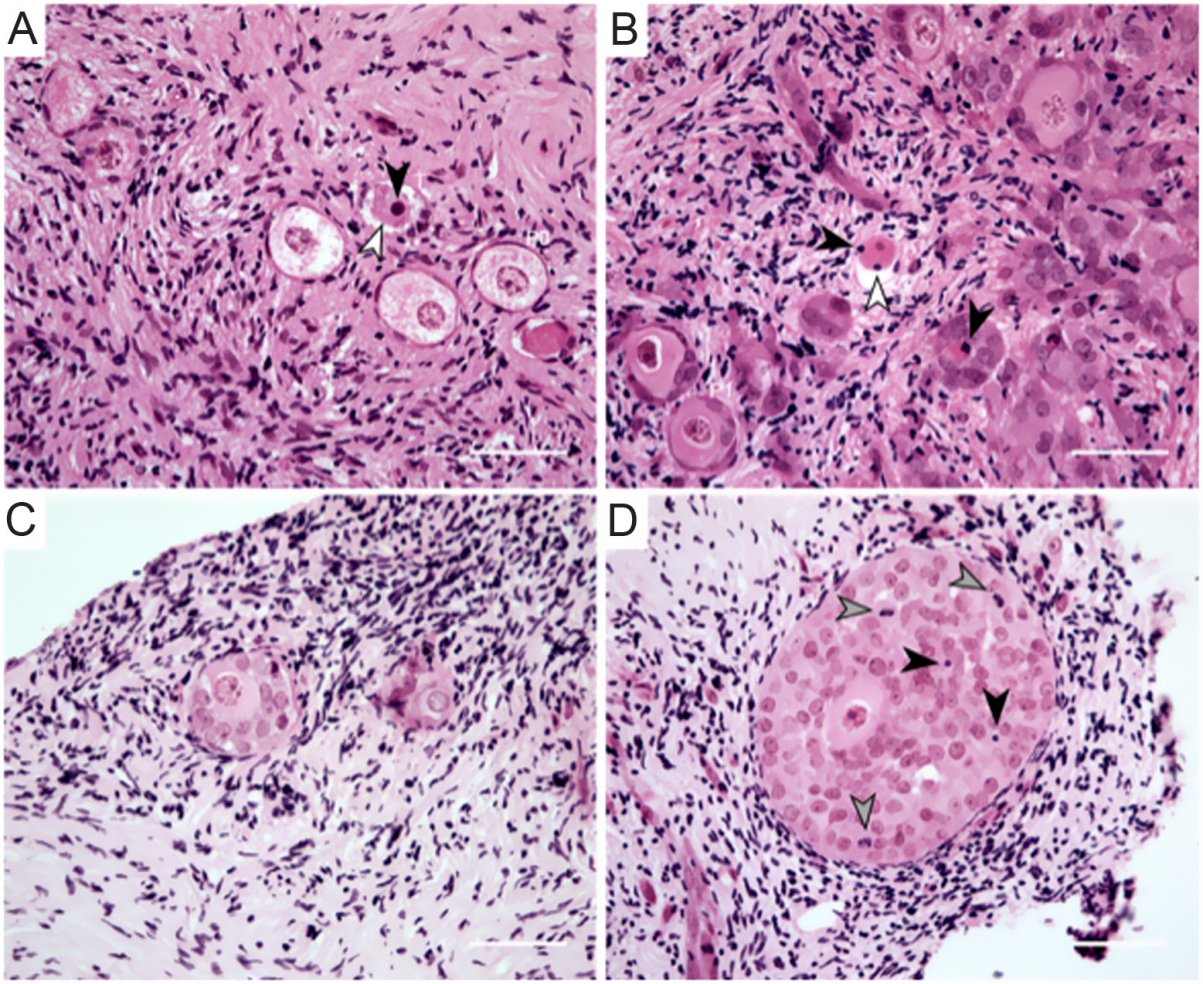
Representative images of non-cultured and cultured cryopreserved human ovarian tissue. Follicles were staged as primordial (P0; single layer of flattened pre-granulosa cells), transitional (T; single layer with at least one cuboidal granulosa cell), primary (P1; single layer of cuboidal granulosa cells) and multilayer (at least one complete layer of granulosa cells plus one or more partial or complete layers). Follicle health was assessed based on the presence or absence of pyknotic granulosa cells or oocyte (black arrowhead) and shrunken ooplasm (white arrowhead). (A) Representative image of non-cultured cryopreserved ovarian tissue from a 22-year-old patient. (B) Representative image of cryopreserved ovarian tissue from a 17-year-old patient cultured for 6 days in a low volume of αMEM medium (AL). (C and D) Examples of normal multilayer follicles after 6 days of culture in AL, same patient as (A). Three granulosa cells undergoing mitosis can be seen in panel D (grey arrowhead). Scale bar = 50 μm.

**Figure 3:**
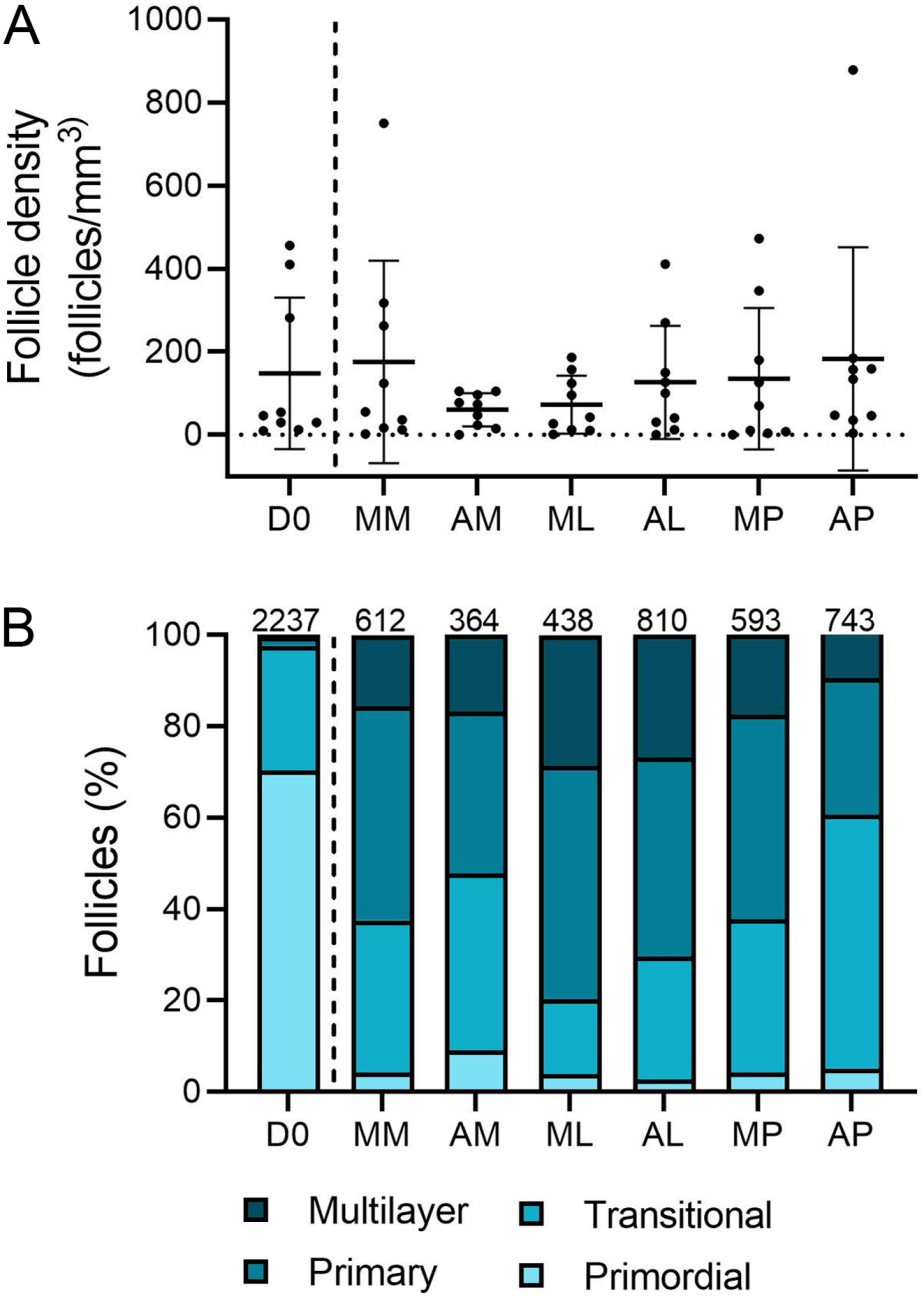
Follicle density and development after 6-day culture of cryopreserved human ovarian tissue. (A) There was no difference in follicle density between any of the culture conditions or non-cultured control. (B) Follicles were staged as primordial (single layer of flattened pre-granulosa cells), transitional (single layer with at least one cuboidal granulosa cell), primary (single layer of cuboidal granulosa cells) and multilayer (at least one complete layer of granulosa cells plus one or more partial or complete layers). Follicle development was observed across all culture conditions compared to non-cultured control (D0, separated by dashed line), with a high proportion of primary and multilayer follicles being observed in cultured samples. The numbers across the top of the columns represent the number of follicles analysed in each group, combined from three patients. D0: non-cultured control; MM: McCoy’s high volume with membrane, AM: αMEM high volume with membrane; ML: McCoy’s low volume; AL: αMEM low volume, MP: McCoy’s high volume, gas permeable plate; AP: αMEM high volume, gas-permeable plate.

**Figure 4:**
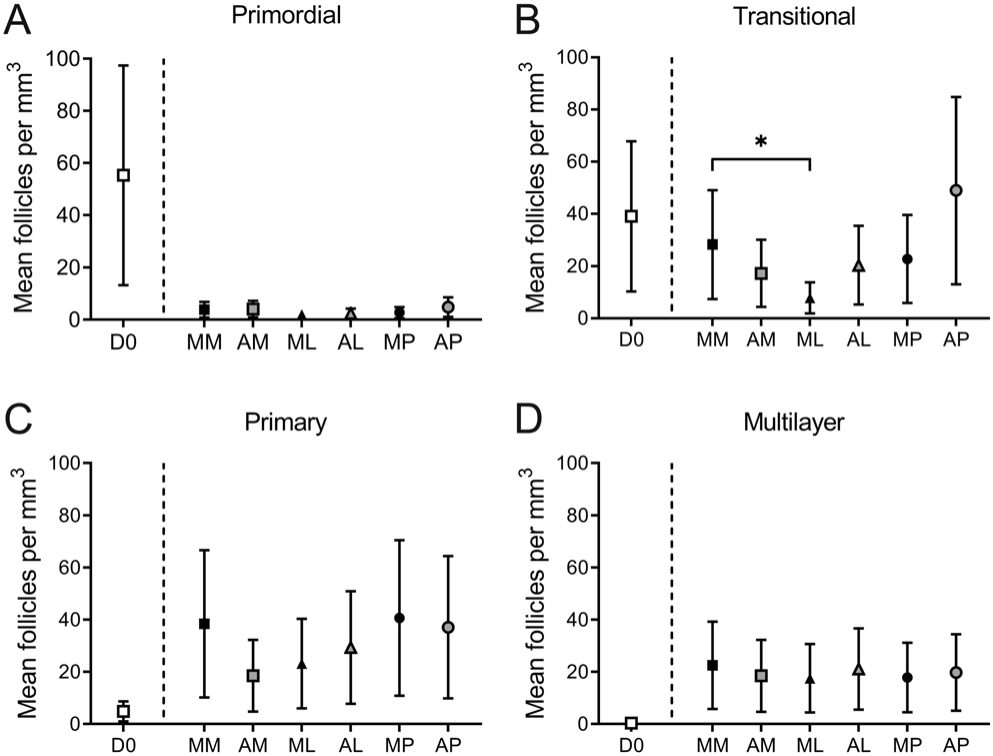
Follicle development is largely unaffected by culture condition. Follicles were classified as (A) primordial, (B) transitional, (C) primary or (D) multilayer based on histology in non-cultured tissue and tissue cultured for 6 days in different conditions. Transitional follicle density was significantly lower in ML compared to MM (*P*  < 0.05). There was no difference in the density of primordial, primary or multilayer follicles between the different conditions tested and MM. Data were analysed using a generalised linear mixed model. *P  < 0.05. D0: non-cultured control; MM: McCoy’s high volume, with membrane AM: αMEM high volume with membrane; ML: McCoy’s low volume; AL: αMEM low volume, MP: McCoy’s high volume, gas-permeable plate; AP: αMEM high volume, gas-permeable plate.

Follicles were classified based on histology as primordial, transitional, primary or multilayer (Fig. 2). There was no difference in follicle density between the different culture conditions (Fig. 3A). Follicles grew during the culture period, with the majority of follicles being classified as primordial (70.4%) or transitional (27.2%) in non-cultured tissue, whereas after 6 days of culture 39.4–79.7% of follicles were at the primary or multilayer stages, depending on the culture condition (Figs 2, 3B and Table 2). Tissue cultured in a low medium volume contained the highest proportion of multilayer follicles, 26.8% for McCoy’s (ML) and 28.5% for αMEM (AL). Follicle development was compared across the different culture conditions using a generalised linear mixed model, with all conditions being compared to culture in a high volume of McCoy’s 5A medium on a polycarbonate membrane (MM) as this was our group’s established culture method based on Lopes *et al.* (2020) prior to this study (Walker *et al.* 2019; Fig. 4). There were significantly fewer transitional follicles in tissue cultured in a low volume of McCoy’s 5A medium (ML) compared to membrane culture (MM; 7.8 ± 6.0 follicles/mm^3^ vs 28.2 ± 20.8 follicles/mm^3^ respectively, *P* <0.05, Fig. 4B). There was no difference between the conditions for follicles at other stages. Follicle density did not affect follicle development (data not shown). Within the multilayer follicle group, only culture in low-volume conditions resulted in follicles with >3 layers of granulosa cells (Table 2). However, there were not enough follicles with ≥3 granulosa cell layers to allow for statistical analysis.

**Table 2:** Proportion of follicles at each developmental stage for non-cultured control (D0) and different culture conditions for human ovarian tissue. Details of the multilayer follicle group are provided, showing the number of follicles with two, three or over three granulosa cell (GC) layers. Data are presented as *n* (%).

Stage	D0	MM	AM	ML	AL	MP	AP
Primordial	1575 (70.4%)	26 (4.2%)	33 (9.1%)	17 (3.9%)	22 (2.7%)	25 (4.2%)	38 (5.1%)
Transitional	609 (27.2%)	204 (33.3%)	141 (38.7%)	72 (16.4%)	218 (26.9%)	199 (33.6%)	412 (55.5%)
Primary	45 (2.0%)	283 (46.2%)	126 (34.6%)	217 (49.5%)	346 (42.7%)	261 (44.0%)	219 (29.5%)
Multilayer	8 (0.4%)	99 (16.2%)	64 (17.6%)	132 (30.1%)	224 (27.7%)	108 (18.2%)	74 (10.0%)
2 GC layers	6 (0.3%)	78 (12.7%)	50 (13.7%)	109 (24.9%)	196 (24.2%)	93 (15.7%)	64 (8.6%)
3 GC layers	2 (0.1%)	21 (3.4%)	14 (3.8%)	21 (4.8%)	27 (3.3%)	15 (2.5%)	10 (1.3%)
≥3 GC layers	0 (0.0%)	0 (0.0%)	0 (0.0%)	2 (0.5%)	1 (0.1%)	0 (0.0%)	0 (0.0%)
Total	2237	612	364	438	810	593	743

AH, αMEM high volume with membrane; AL, αMEM low volume; AP, αMEM high volume, gas-permeable plate; ML McCoy’s low volume; MM, McCoy’s high volume with membrane; MP, McCoy’s high volume, gas permeable plate.

### Culture in low volume conditions improved follicle morphology

**Figure 5:**
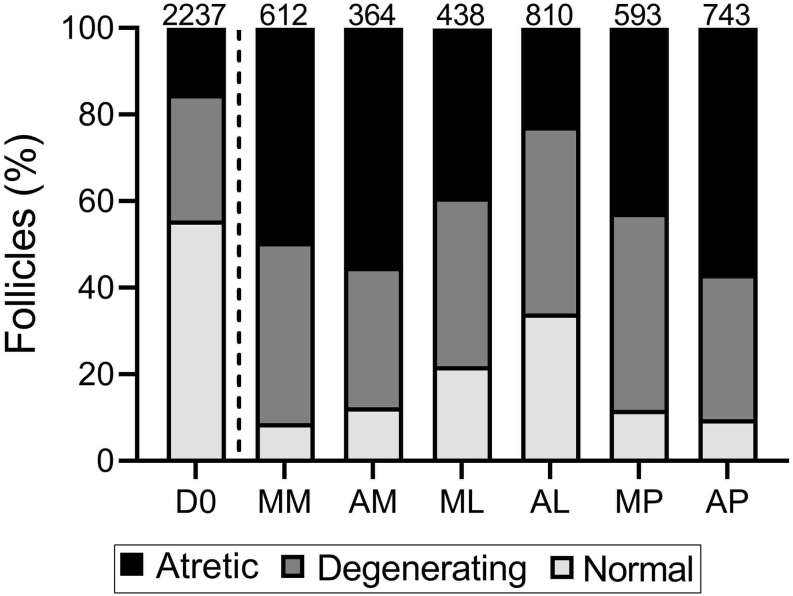
Follicle morphological health after 6-day culture of cryopreserved human ovarian tissue. Follicles were classified as morphologically normal (no evidence of degeneration), degenerating (presence of either a pyknotic oocyte, pyknotic granulosa cells or shrunken ooplasm), and atretic (presence of both pyknotic oocyte and pyknotic granulosa cells). The proportion of atretic follicles was increased in cultured tissue compared to non-cultured control. The numbers across the top of the columns represent the number of follicles analysed in each group, combined from three patients. D0: non-cultured control; MM: McCoy’s high volume with membrane, AM: αMEM high volume with membrane; ML McCoy’s low volume; AL: αMEM low volume, MP: McCoy’s high volume, gas permeable plate; AP: αMEM high volume, gas-permeable plate.

**Figure 6:**
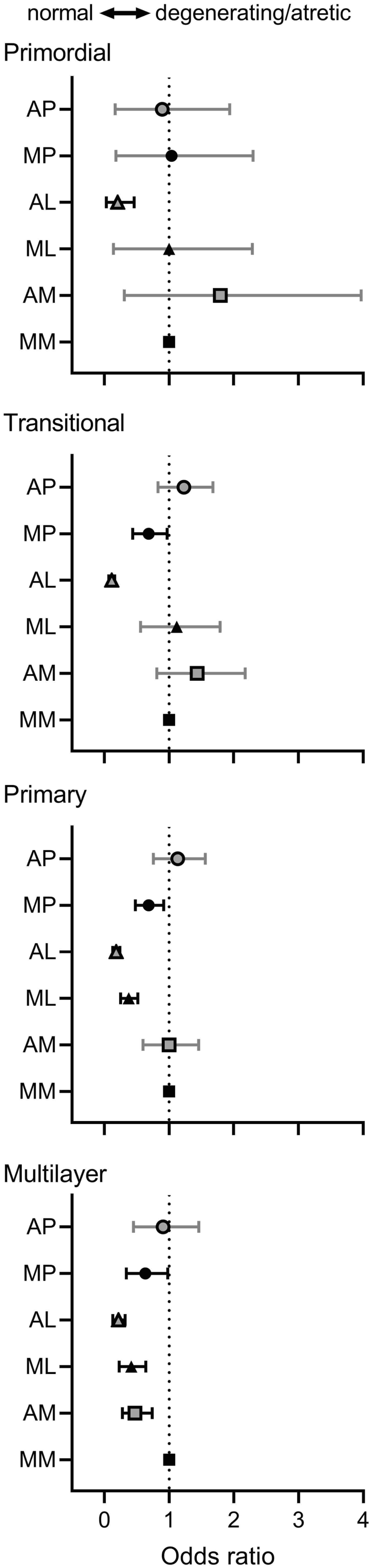
Effect of culture condition on follicle morphological health. A proportional odds ratio model was used to determine whether any of the conditions tested was more likely to lead to morphologically healthier follicles at each stage compared to MM. Data are represented as odds ratio with upper and lower confidence intervals (displayed as error bars). Odds ratio < 1 indicates decreased odds of a follicle being degenerating or atretic compared to MM. Odds ratio > 1 indicates increased odds of a follicle being degenerating or atretic compared to MM. Those conditions where confidence intervals do not cross 1 (black lines) were significantly less likely to have degenerating or atretic follicles compared to the baseline (MM, *P* < 0.05). MM: McCoy’s high volume with membrane, AH: αMEM high volume with membrane; ML McCoy’s low volume; AL: αMEM low volume, MP: McCoy’s high volume, gas permeable plate; AP: αMEM high volume, gas-permeable plate.

Follicles were classified as morphologically normal, degenerating or atretic. Non-cultured tissue contained mostly normal follicles at all stages, however, after 6 days of culture the proportion of degenerating or atretic follicles had increased considerably for all culture conditions (Figs 2 and  5). As with development, follicle morphological health was not affected by follicle density (data not shown). A proportional odds model was used to determine whether a follicle was more likely to be normal compared to degenerating or atretic after 6 days in a particular culture condition (Fig. 6). Compared to MM, tissue cultured in low volume conditions (McCoy’s, ML or αMEM, AL) or permeable dish conditions (MP or AP) had greater odds of normal multilayer follicles being observed compared to degenerating or atretic (Fig. 6D). The most marked improvement in the morphology of multilayer follicles was seen in the low volume conditions, particularly AL where the odds of observing a normal follicle were five times greater than MM (OR = 0.2; 95% CI 0.13–0.32; *P* < 0.001). Tissue cultured in ML had 2.6 times greater odds of multi-layered follicles being classified as ‘normal’ follicles vs ‘degenerating’ or ‘atretic’ compared to MM (OR = 0.38; 95% CI 0.23–0.64; *P* < 0.001).

As culture in AL resulted in morphologically healthier follicles of all stages compared to MM, AL was set as the baseline level of comparison in the proportional odds model, to ascertain whether medium affected follicle health within the low volume condition. Compared to AL, multilayer follicles cultured in ML had 1.9 times greater odds of being classified as degenerating or atretic than normal (OR = 1.92; 95% CI 1.24–2.97; *P* < 0.001) showing that AL yielded superior follicle morphology compared to the other conditions tested (Fig. 2). When the proportion of normal primary or multilayer follicles was compared between culture conditions, we observed that culture in AL yielded over 20% normal primary or multilayer follicles, more than the other culture conditions.

## Discussion

Here we report that culture medium and plate conditions significantly impact the morphology of follicles generated from 6-day *in vitro* culture of cryopreserved human ovarian tissue. We found that culture in a low volume of αMEM resulted in morphologically healthier follicles at all stages of development compared to the same volume of McCoy’s 5A medium, and culture using polycarbonate membranes or gas-permeable plates with a higher medium volume.

Our results demonstrate that culture in a low medium volume led to improved follicle morphology compared to an approximately three-fold higher medium volume with a polycarbonate membrane or a gas-permeable dish. It should be noted that all three plate conditions (polycarbonate membrane, low volume and gas-permeable dish), all provided equal access to the air as the ovarian tissue was located close to the medium-gas interface (Fig. 1). Improved follicle morphology in low volume culture may have been due to higher concentrations of soluble secreted factors released by follicles or their surrounding cells which may have contributed to decreased follicle atresia. By culturing cryopreserved ovarian tissue in a low volume of αMEM we observed a high level of follicle progression, with 26.8% multilayer follicles (two or more partial or complete granulosa cell layers) out of 810 follicles from three patients, with 34.0% morphologically normal follicles. Oxygen availability has previously been demonstrated to be a contributing factor to follicle health (Morimoto *et al.* 2007, Talevi *et al.* 2018). Talevi *et al.* (2018) reported that culture of fresh human ovarian tissue in 5 mL of αMEM medium (column height 1.4 mm) in a gas-permeable petri dish led to improved follicle development and health, reporting that out of 287 follicles from six patients, 19.5% were multilayer and 41.7% normal after 6 days of culture. This culture method is similar to that of a low volume of αMEM (AL) described in the current study (AL column height 1.6 mm), however the medium volume used by Talevi *et al.* (2018) was 15 times greater. Taking into account the potential benefits of low volume conditions mentioned above, culturing tissue in a low volume of αMEM in gas-permeable plates may lead to even greater follicle health and should be further investigated.

Culture in a low medium volume (ML and AL) yielded the highest proportion of normal primary and multilayer follicles out of the six conditions tested. Our culture in a low volume of McCoy’s 5A is similar to the first of a four-step culture method used by McLaughlin *et al.* (2014), the only successful generation of mature human oocytes grown from ovarian tissue (McLaughlin *et al.* 2018), however, we found that culture in a low volume of αMEM resulted in superior follicle morphological health at all stages of development. Here, ovarian tissue was cultured for 6 days, which has previously been reported to support follicle development (Telfer *et al.* 2008), with a similar proportion of secondary follicles being reported after 6 and 9 days of culture (Talevi *et al.* 2018). This short-term culture is the initial step for IVG and the most limiting since it determines the number of follicles available for subsequent culture. Therefore, improving the health and development of early growing follicles could improve the yield of isolated follicles for IVG.

Studies describing culture of controlled-rate cryopreserved human ovarian tissue, as we have used in the present study, are limited, and the majority of studies use fresh ovarian tissue (Telfer *et al.* 2008, McLaughlin *et al.* 2018, Talevi *et al.* 2018, Lopes *et al.* 2020). Follicle growth *in vitro* may be compromised by cryopreservation and cryopreserved ovarian tissue may have different requirements *in vitro*, as has been demonstrated using animal models (Ting *et al.* 2011, Castro *et al.* 2014). Interestingly, Castro *et al.* (2014) reported improved follicle viability in cryopreserved bovine ovarian tissue after culture in McCoy’s 5A medium compared to αMEM and M199, which is in contrast with the results presented here where αMEM was found to be superior. However, the culture systems cannot be directly compared, as Castro *et al.* (2014) cultured samples under 1 mL of medium in conventional 24-well plates, therefore oxygen availability was different to the culture systems described here, where all tissue was at the medium-gas interface. αMEM has also found to be superior for culture of fresh bovine and human ovarian tissue (Wright *et al.* 1999, Jimenez *et al.* 2016), highlighting the need to optimise ovarian tissue culture for both fresh and cryopreserved tissue. This is indeed critical since there are many patients who already have tissue cryopreserved and may require IVG to generate eggs.

Chemotherapy treatment prior to ovarian tissue cryopreservation may also compromise the development of these follicles *in vitro*, however only two studies exist and data are confounded by differences in the age between control and treated groups (Asadi Azarbaijani *et al.* 2015, Pampanini *et al.* 2019). Clearly, if we are to progress to a clinical treatment, the effect of prior chemotherapy on subsequent follicle development *in vitro* needs to be explored.

Follicles at all stages were more likely to be graded as normal when cultured in a low volume of αMEM (AL) compared to the same volume of McCoy’s 5A medium (ML). αMEM contains both sodium pyruvate and a physiological concentration of glucose (5.6 mM), whereas McCoy’s 5A contains no pyruvate and three times higher concentration of glucose (16.7 mM) and therefore αMEM may provide a more suitable environment for follicle metabolism. Previous studies have indeed demonstrated pyruvate to be the main energy source during early follicle development in mice (Harris *et al.* 2007, 2009). Under normal conditions, the oocyte is supplied with pyruvate by its supporting somatic cells however it has been demonstrated that pyruvate is taken up from the culture medium during *in vitro* follicle culture (Harris *et al.* 2009). Therefore, αMEM may provide a more suitable energy source for early follicle metabolism compared to McCoy’s, thereby better supporting follicle health although it is unknown at this time if cryopreservation has any effect on subsequent tissue requirements in culture.

The analysis presented in the present study was performed based on clearly defined and detailed morphological criteria, as previously described (Walker *et al.* 2019). While morphology is a widely used and generally accepted measure of follicle health and survival (McLaughlin *et al.* 2014, Talevi *et al.* 2018, Pampanini *et al.* 2019, Walker *et al.* 2019, Lopes *et al.* 2020), there are some limitations in using morphological parameters alone to evaluate follicle health. Namely, fixatives such as neutral buffered formalin cause tissue-dependant shrinkage, which we have observed can obscure the morphology of human ovarian follicles in particular, and may result in misinterpretation of data (Adeniran *et al.* 2021). In the present study, tissue was fixed in Bouin’s solution, which prevents tissue shrinkage and better preserves morphology, however, this fixative is incompatible with molecular analysis such as immunohistochemistry. Due to the limited availability of cryopreserved ovarian tissue and the heterogeneity of follicles within the tissue, we focussed solely on well-defined morphological outcome measures for the present study. In studies using a combination of morphological and molecular analyses, the two methods are generally in agreement (Talevi *et al.* 2018). Indeed, molecular analysis may present its own set of limitations, including challenges in selecting an appropriate marker in addition to the transience of some marker expression (Pampanini *et al.* 2019).

In conclusion, we report that culture in a low volume of αMEM in conventional 24-well plates led to improved follicle health after 6 days of culture compared to other conditions with similar oxygen availability. This highlights the need to further optimise culture systems for ovarian tissue culture, particularly culture of cryopreserved ovarian tissue. Improving the health and early development of follicles *in vitro* is a key aspect of developing culture systems capable of supporting follicle development from the earliest stages to result in mature fertilisable eggs. Once developed, these methods could offer a significant number of individuals a chance of achieving pregnancy following cancer treatment.

## Declaration of interest

Suzannah Williams is a Lay Editor of Reproduction and Fertility. Suzannah Williams was not involved in the review or editorial process for this paper, on which he/she is listed as an author

## Funding

This work was supported by the Oxford Medical Research Council http://dx.doi.org/10.13039/501100000265 Doctoral Training Programme (Oxford MRC-DTP) grant awarded to B D B (grant number MR/N013468/1) and by a joint scholarship to C A W from the Clarendon fund and NDWRH.

## Author contribution statement

S A W, C A W and B D B designed the study. M F obtained the original ethical approval which was modified by S A W and approved by M F. S L was instrumental in obtaining the tissues used in the study and is a supervisor of B D B. C A W and B D B carried out all cultures and data analysis. C A W, B D B and S A W wrote and prepared the manuscript. All authors critically read and approved the final manuscript.
